# Lower methane emissions were associated with higher abundance of ruminal Prevotella in a cohort of Colombian buffalos

**DOI:** 10.1186/s12866-020-02037-6

**Published:** 2020-11-27

**Authors:** Sandra Bibiana Aguilar-Marin, Claudia Lorena Betancur-Murillo, Gustavo A. Isaza, Henry Mesa, Juan Jovel

**Affiliations:** 1 Facultad de Ciencias Agropecuarias, Universidad de Caldas, Caldas, Colombia; 2 Escuela de Ciencias Básicas, Tecnología e Ingeniería, Universidad Nacional Abierta y a Distancia, UNAD, Bogotá, Colombia; 3 Departamento de Sistemas e Informática - Facultad de Ingenierías, Universidad de Caldas, Caldas, Colombia; 4 Office of Research. Faculty of Medicine and Dentistry, University of Alberta, Edmonton, AB T6G 2E1 Canada

**Keywords:** Methane emissions, Methanogenesis, *Prevotella* abundance, Propionic acid synthesis

## Abstract

**Background:**

Ruminants burp massive amounts of methane into the atmosphere and significantly contribute to the deposition of greenhouse gases and the consequent global warming. It is therefore urgent to devise strategies to mitigate ruminant’s methane emissions to alleviate climate change. Ruminal methanogenesis is accomplished by a series of methanogen archaea in the phylum Euryarchaeota, which piggyback into carbohydrate fermentation by utilizing residual hydrogen to produce methane. Abundance of methanogens, therefore, is expected to affect methane production. Furthermore, availability of hydrogen produced by cellulolytic bacteria acting upstream of methanogens is a rate-limiting factor for methane production. The aim of our study was to identify microbes associated with the production of methane which would constitute the basis for the design of mitigation strategies.

**Results:**

Moderate differences in the abundance of methanogens were observed between groups. In addition, we present three lines of evidence suggesting an apparent higher abundance of a consortium of *Prevotella* species in animals with lower methane emissions. First, taxonomic classification revealed increased abundance of at least 29 species of *Prevotella*. Second, metagenome assembly identified increased abundance of *Prevotella ruminicola* and another species of *Prevotella*. Third, metabolic profiling of predicted proteins uncovered 25 enzymes with homology to *Prevotella* proteins more abundant in the low methane emissions group.

**Conclusions:**

We propose that higher abundance of ruminal *Prevotella* increases the production of propionic acid and, in doing so, reduces the amount of hydrogen available for methanogenesis. However, further experimentation is required to ascertain the role of *Prevotella* on methane production and its potential to act as a methane production mitigator.

**Supplementary Information:**

The online version contains supplementary material available at 10.1186/s12866-020-02037-6.

## Background

Greenhouse gases include carbon dioxide (CO_2_), methane (CH_4_), nitric oxide (N_2_O) and ozone (O_3_) [1]. The atmospheric content of CO_2_ and CH_4_ has increased dramatically since the industrial revolution [1]⁠ and are major contributors to global warming [2]⁠. It has been estimated that methane may constitute up to 20% of greenhouse gases [3]⁠ and wetland and ruminant methane emissions have been on the rise since 2007, mainly in tropical and subtropical regions [4]⁠.

Livestock production systems may account for up to 14% of all anthropogenic methane emissions [5]⁠. Consequently, more than 100 countries committed, in the Paris agreement of 2015, to reduce greenhouse emissions from agricultural activities [6]⁠. In order to achieve this, however, a full understanding of methanogenesis and its associated microbes is needed. Methane is produced during enteric fermentation in ruminants by anaerobic microorganisms collectively known as methanogens in the Archaea domain and the phylum Euryarchaeota [7]⁠. Plants fix atmospheric CO_2_ through photosynthesis and generate biomass rich in carbohydrates that is used to feed ruminants. Polysaccharides digestion then takes place under anoxic conditions in the rumen and hindgut [7]⁠ and includes a complex of anaerobic bacteria, fungi and protozoa that progressively process carbohydrates through hydrolysis and fermentation to produce acetic acid, CO_2_ and H_2_. Fermentation also generates short-chain fatty acids, like acetate, propionate and butyrate, which constitute an energy source for many cell metabolic processes and contribute to homeostasis of the digestive system [8]⁠. Finally, methanogens convert CO_2_ and H_2_ to methane via the hydrogenotrophic pathway. Alternatively, the methylotrophic pathway produces methane using methylamines and methanol as substrates [9]⁠. Methanogenesis is considered an essential process for ruminants because if hydrogen generated during carbohydrate fermentation is not removed, it may inhibit microbiome metabolism [7]⁠. Finding ways to redirect hydrogen metabolism is a promising avenue to mitigate methane emissions and also to improve energy retention from grazing [10]⁠.

Changes in abundance of methanogens themselves would definitely affect methane production, but it remains also possible that changes in microbial composition and structure that result in perturbation of hydrogen metabolism or accumulation may also impact methanogenesis [11]⁠. Indeed, application of candidate hydrogenotrophic bacteria that could redirect hydrogen away from methanogenesis has been proposed as a strategy to mitigate methane emissions [10]⁠. More complex relationships are also possible. For example, it has been proposed that not only the abundance of methanogens but also the composition of methanogenic communities in the rumen seems to exert a strong effect on methane emissions. Namely, the presence of species within the *Methanobrevibacter gottschalkii* clade has been reported associated with higher production of methane [12]⁠. Multiple factors including breed, sex and diet, affect microbiome composition [13, 14]⁠, and host genetics play a significant role in methane emissions without influencing microbiome composition [15]⁠.

The buffalo rumen microbiome remains largely underexplored. In preliminary studies, the rumen microbiome of Surti and Mehsani buffalos was found to be dominated by phylotypes belonging to the Bacteroidetes/Chlorobi, Firmicutes and Protobacteria phyla, and the metagenome was consistent with a genetic profile specialized in carbohydrate fermentation [16]⁠. Although in a very small cohort, Kala and collaborators reported that bacteria in the genera *Prevotella*, *Bacteroides*, *Clostridium*, *Ruminococcus*, *Eubacterium*, *Parabacteroides*, *Fibrobacter* and *Butyrivibrio* were the most abundant inhabitants of the buffalo rumen, and that the abundance of *Ruminococcus flavefaciens* and *R. albus* increased when animal were fed with high-roughage diet [17]⁠. Finally, it was found that abundances of individual taxa and specific metabolites were correlated. For instance, *Acetobacter* abundance was positively correlated with acetate, propionate and butyrate content, and so was *Prevotella* abundance and butyrate content [18]⁠. Thus, an important question that remains largely unanswered is how changes in microbiome structure affect methanogenesis. Clearly, more studies on the rumen microbiome in different breeds and geographical regions are urgently needed.

We conducted a microbiome survey in ruminal fluids of two cohorts of buffalos from a dairy farm in the department of Cordoba, Colombia, which were shown to produce low or high methane emissions. We hypothesized that animals with greater emissions of methane contained more abundant methanogenic microbes. However, our study revealed that the overall microbial composition did not appear very different among groups; instead, we detected higher abundance of *Prevotella* in the group with lower methane emissions. An hypothetical scenario to explain the inverse correlation between *Prevotella* abundance and methane emissions is presented.

## Results

**Fig. 1:**
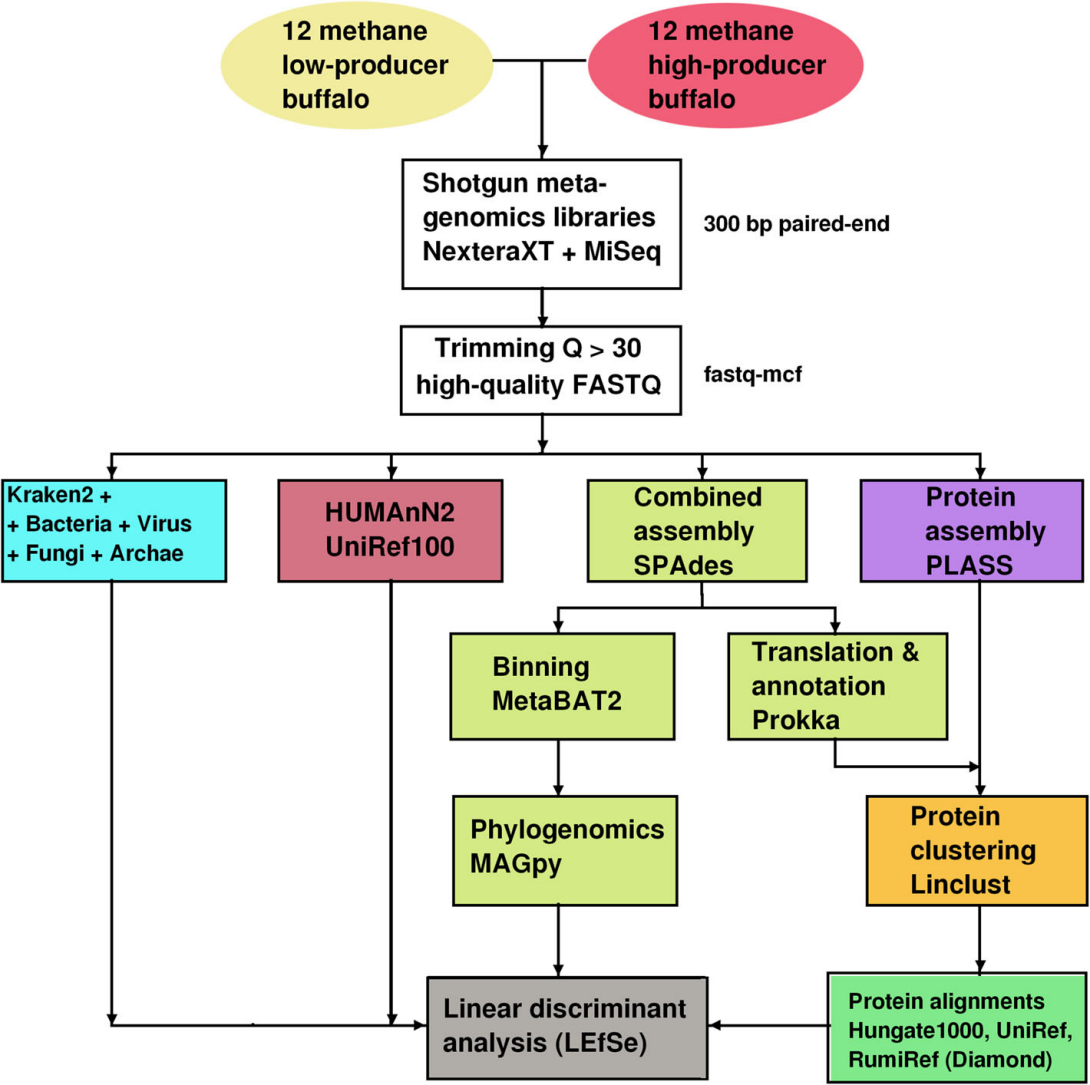
Bioinformatics pipeline used for data analysis. Twelve buffaloes were included in each group. After quality control, individual sequences were taxonomically classified with Kraken2 and functionally analyzed with HUMAnN2. Combined assembly was conducted with SPAdes and assembled contigs were annotated with Prokka. In parallel, binning of contigs was conducted with MetaBAT2 and such bins were phylogenetically analyzed with MAGpy. De novo assembly of proteins was carried out with PLASS. Protein sequences annotated with Prokka or assembled with PLASS were consolidated and clustered with Linclust to determine a set of non-redundant representative sequences, which were aligned against several protein databases using Diamond. All statistical comparisons were conducted with LEfSe. A description of all command lines used is in the bitbucket repository (https://github.com/buffGenomic/PipelineColBuff)

With the advent of next generation sequencing (NGS), the microbiome of ruminants is being profiled at high resolution and throughput and a complex picture is emerging wherein host genetics and microbiome structure additively contribute to several phenotypes, including methane emissions [13]⁠. In order to investigate the ruminal microbiome composition of two cohorts of Colombian buffalos found to produce high or low levels of methane, we conducted shotgun metagenomics. The bioinformatics pipeline used in this study is described in Fig. 1.

### Alignment of individual sequences

 Taxonomic classification of sequences was performed using the software Kraken2 [19]⁠, and the standard database complemented with all bacterial, fungal, viral and archaeal sequences in the GenBank (including incomplete genomes) plus all sequences deposited in the Genome taxonomy database, GTDB (gtdb.ecogenomic.org). The GTDB hosts 145,512 bacterial accessions and 2392 archaeal accessions [20]⁠. We discarded Kraken2 hits with less than 10% of the k-mers matching the reference sequence. Then, we kept hits with a relative abundance of at least 3 reads (sequences) per sample. A list of hits obtained with Kraken2 pseudoalignments is presented in Supplementary Table S1. A total of 582 taxa were identified in our data. Five taxa corresponded to Archaea (Supplementary Fig. S1), along with 576 taxa in the domain Bacteria, one fungus of unknown taxonomy and two virus taxa of unknown taxonomy.

**Fig. 2:**
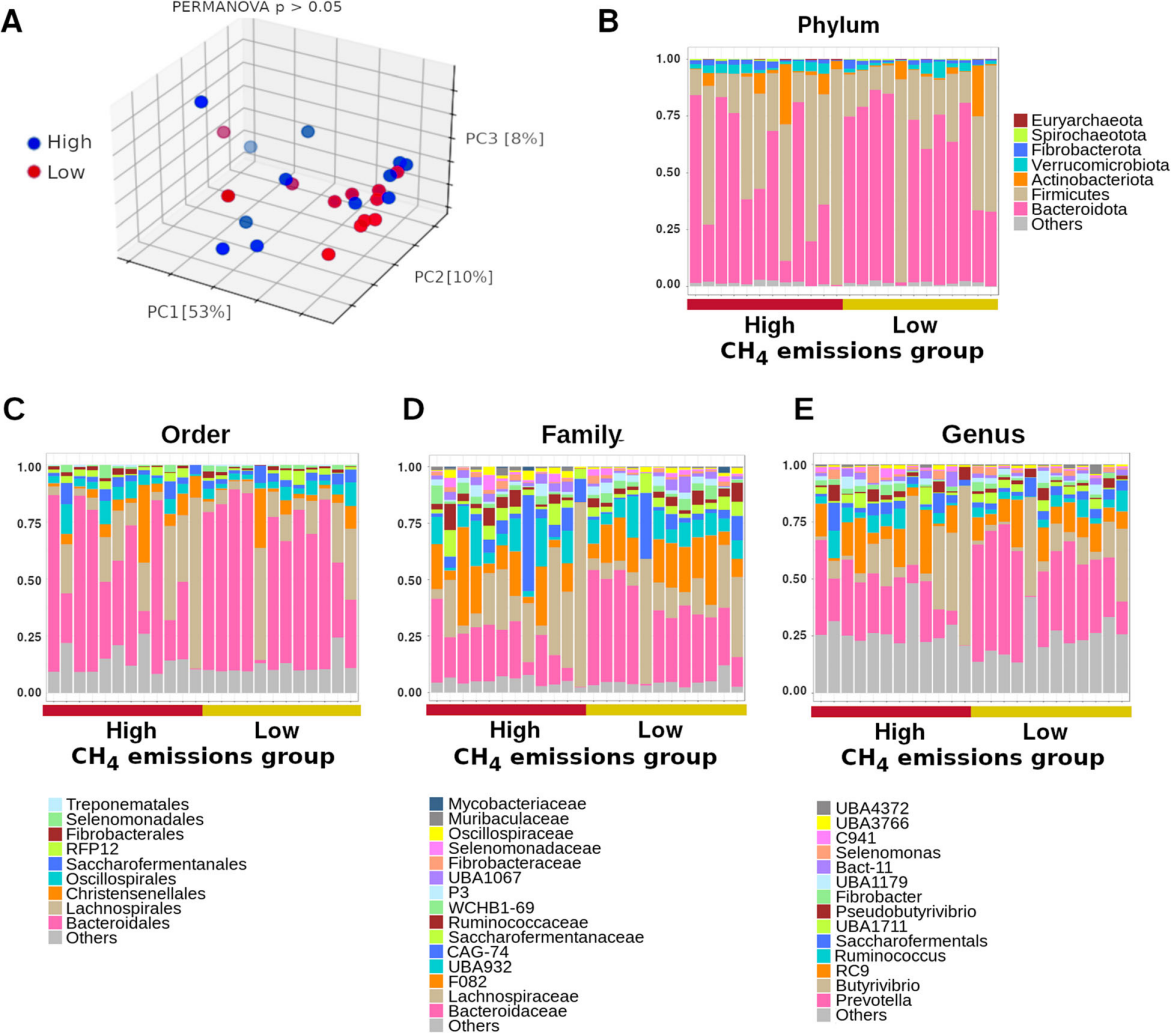
Characterization of the rumen microbiome in buffalo cohorts. **a** Principal coordinate analysis plot. Permanova analysis between groups showed a non-significant *p*-value. Taxonomic classification of sequences at the phylum (**b**), order (**c**), family (**d**), and genus (**e**). *Prevotella* or upper taxa containing it are in pink colors in panels **b-e**

Principal coordinate analysis ordination of a Bray-Curtis dissimilarity matrix showed that the microbiome of both groups of buffalos is apparently different, although there is considerable variability among animals in each group (Fig. 2a). The PCoA plot depicted in Fig. 2a shows that, along the first component (PC1), which capture 53% of the variance, most blue points (high methane emissions) located on the left part of the plot, while most red points (low methane emissions) clustered on the right part of the plot. Permutational Analysis of Variance (PERMANOVA), however, did not detect statistically significant differences between groups. We also conducted Analysis of Similarity (ANOSIM) and obtained a significant *p*-value (0.02), which again suggests that the groups under comparison are not statistically different. At the phylum level, the microbiome was dominated by Bacteroidetes and Firmicutes, and in decreasing abundance Actinobacteria and Protobacteria. Euryarchaeota was among the seven most abundant phyla, but with abundance much lower than that the rest of phyla (Fig. 2b). At the genus level, *Prevotella* was somewhat more abundant in the group of animals with lower methane emissions (Fig. 2e). At higher taxonomic levels, family, order and phylum, the same subtle trend was observed for Bacteroidaceae, Bacteroidales and Bacteroidota, respectively, which showed a moderately higher abundance in the group with lower methane emissions. However, high variability is evident inside each group.

**Fig. 3:**
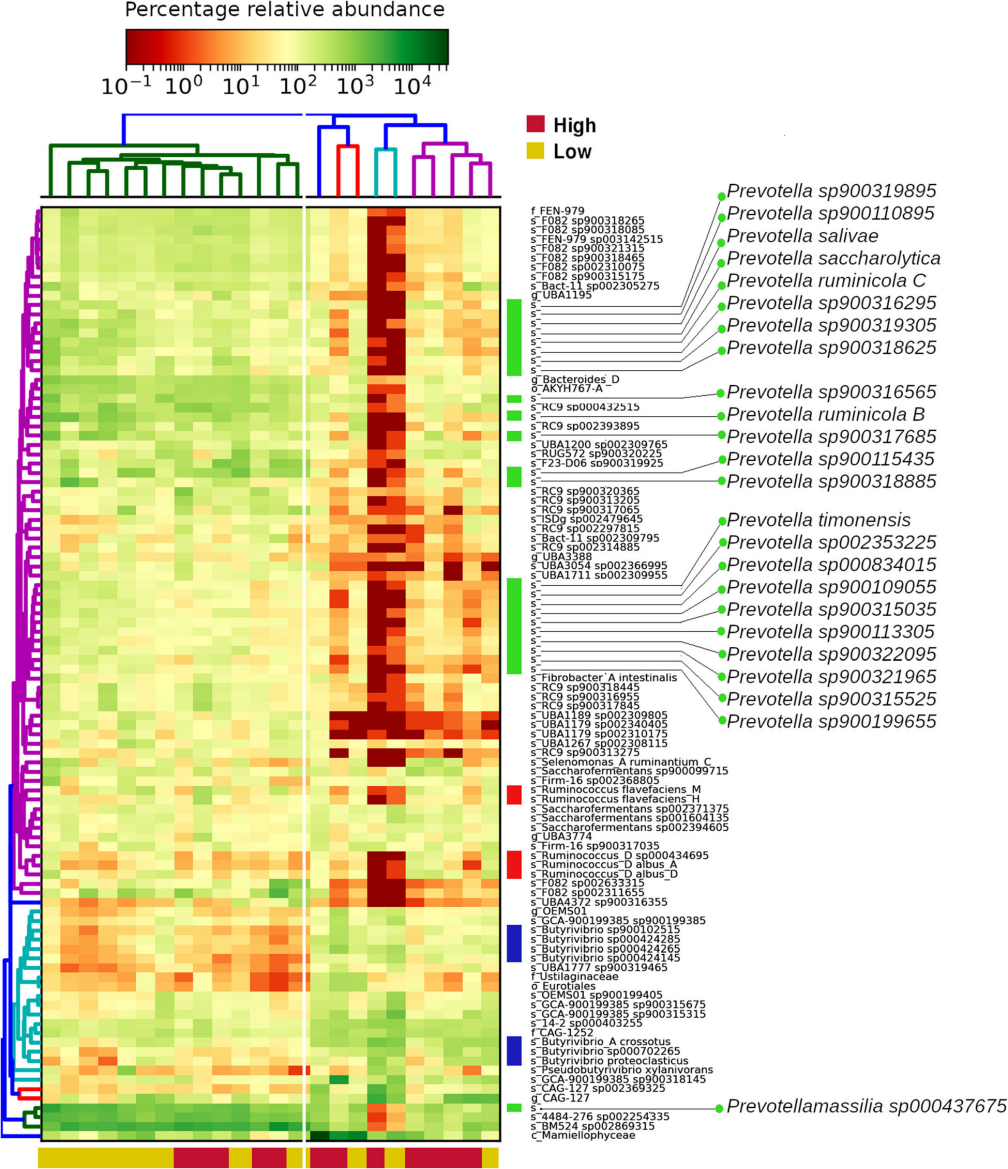
Hierarchical clustering of the 100 most abundant taxa using Bray-Curtis dissimilarities and the hclust method. Green bars on the right side of the heatmap indicate *Prevotella* species. Red bars indicate *Ruminococcus* species. Blue bars indicate *Butyrivibrio* species

We subjected the 100 most abundant taxa to hierarchical clustering of their Bray-Curtis dissimilarity indices using the hclust algorithm, which led to the identification of two clusters (Fig. 3). Namely, a larger cluster comprising 14 samples was integrated by 9 animals (64%) from the low-methane-emissions group and 5 animals (36%) from the high-methane-emissions group. This group exhibited higher abundance of the majority of the 100 most abundant bacteria, including 23 species of *Prevotella* (green bars), seven species of *Butyrivibrio* (blue bars), five species of *Ruminococcus* (red bars), also *Selenomonas ruminantium*, and *Fibrobacter intestinalis*, among others. The other cluster (on the right), was more heterogeneous and contained mostly animals from the high-methane-emissions group.

We compared the abundance of the five taxa in the family *Methanobacteriaceae* detected in our libraries. In general, a subtle higher abundance in four out of five *Methanobacteriaceae* taxa in the group of animals with higher methane emissions was observed (Supplementary Fig. S1). Such differences did not reach statistical significance, but they are clearly appreciated in the boxplots, although considerable variability inside groups was also observed (Supplementary Fig. S1).

**Fig. 4:**
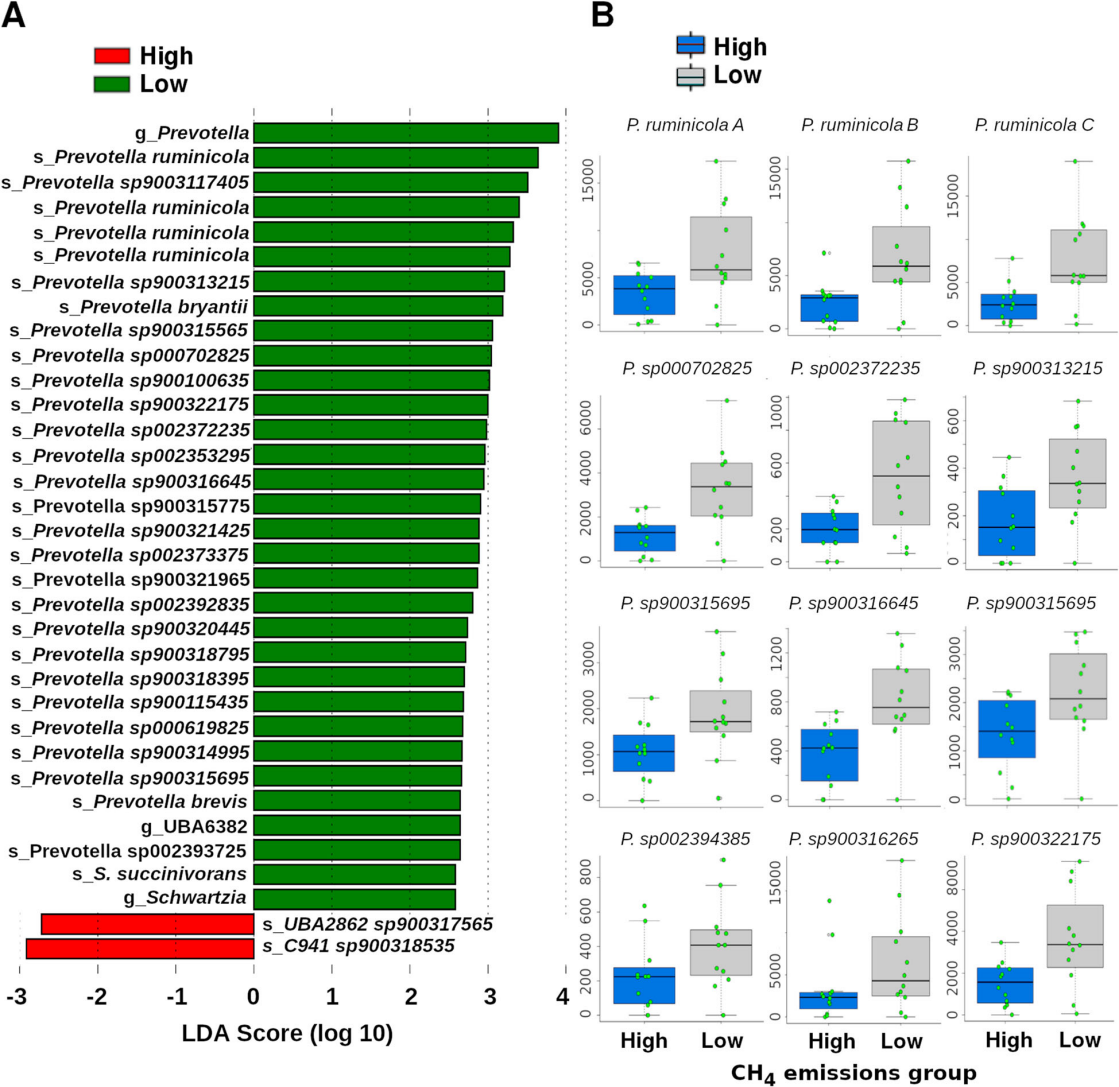
Statistical comparison of taxa. **a** Linear discriminant analysis results. LEfSe was run with default parameters. **b** Box plots presenting the relative abundance of 12 *Prevotella* species in samples associated with high or low methane emissions

We subjected the abundance of all taxa detected to linear discriminant analysis, using the software LEfSe [21]⁠. Interestingly, based on uncorrected *p*-values, LEfSe suggested that at least 29 species in the genus *Prevotella* were more abundant in the low-methane-emissions group (Fig. 4a). However, although LEfSe reported increased abundance of those *Prevotella* species, in most cases p-values lost significance after correction for multiple comparisons with the Benjamini-Hochberg method. To better characterize this observation, we plotted the relative abundance of twelve species of *Prevotella* (Fig. 4b) and a clear trend is observed, although variability inside each group is considerably large.

We also conducted metabolic profiling with HUMAnN2 [22], but as is usual with non-human samples, results were incomplete and not very informative.

### Combined assembly and identification of bacterial genomes

NGS libraries are a very fragmentary representation of the metagenome [23]⁠. Thus, recovery of bacterial genomes is incomplete and somewhat stochastic. This implies that in two or more samples harbouring the same bacterium, different parts of the genome might be recovered. Therefore, it makes sense to conduct sequence assembly combining all samples and after identification/annotation of assembled contigs, alignment of individual samples to assembled contigs will determine the relative contribution, if any, of each sample to each contig. Therefore we conducted combined assembly of all 24 samples. The assembly of reads generated 3.6 million contigs with an N50 of 481 bp (min. 373; max. 53,776; average 491). We then subjected such contigs to binning with metaBAT2 [24]⁠. From those, MetaBAT2 was able to cluster 78 putative bacterial genomes (bins), which were subjected to phylogenetics analysis and annotation with MAGpy [25]⁠. Hereinafter, bins are referred to as MAGs.

**Fig. 5:**
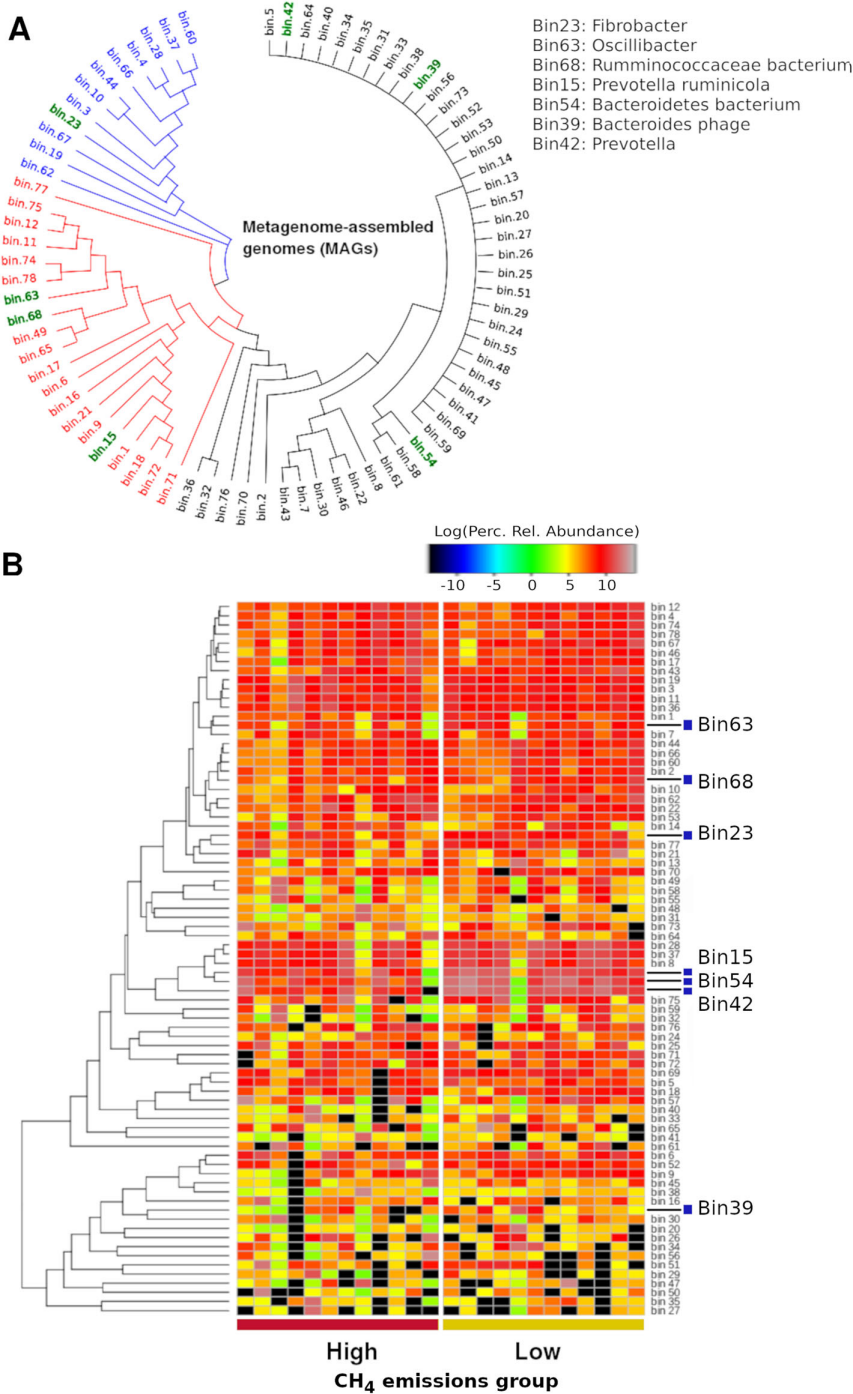
Metagenome assembled genomes (MAGs). **a** Phylogenetic tree generated by MAGpy. **b** Relative contribution of each sample to each putative genome

The phylogenetic tree generated by MAGpy comprised three major clusters. The larger cluster (Fig. 5a; in black) contained many putative genomes that were very similar among them. The other two branches of the phylogenetic tree were more heterogeneous. The annotation assigned to each bin can be found in Supplementary Table S2. LEfSe analyses suggested that the genera *Fibrobacter*, *Oscillibacter*, *Prevotella* and the species *Ruminococcaceae bacterium*, *Prevotella ruminicola*, *Bacteroidetes bacterium* and a phage from Bacteroidetes were more abundant in the low-methane-emissions group, but *p*-values lost significance after correction for multiple comparisons. Because contigs used in this study are the result of an assembly procedure that included all samples, the relative contribution of each sample to each MAG is presented in Fig. 5b. Essentially, most MAGs were represented in all samples, with rather few exceptions (black cells in the lower half of heat map). The relative abundance of MAGs reported above is visibly larger in the group with lower methane emissions, but considerably heterogeneity is observed inside groups.

### Protein predictions and annotation

We predicted a total of 4,467,657 representative non-redundant protein sequences that were aligned against the protein databases UniRef100 [26]⁠, RumiRef100 [26]⁠ and Hungate1000 [27]⁠. RumiRef and Hungate1000 are databases of bacterial sequences from ruminal samples. Alignments were conducted with Diamond and the top hits were recovered. The best hit from each of the three alignments was selected for annotation.

To quantify the contribution of each sample to each protein sequence, individual samples sequences were aligned to the protein reference sequences with the method blastx of Diamond. An astringent filtering procedure was implemented as described in the Methods section. A total of 1309 protein sequences passed such filtering process (Supplementary Table S3). Among proteins most frequently detected were FAD-dependent oxidoreductase, Acyl-CoA dehydrogenase, Urocanate hydratase (homologous to *Prevotella)*, subunit beta of a DNA-directed RNA polymerase, Methylmalonyl-CoA mutase, Pyridine nucleotide-disulfide oxidoreductase (homologous to *Eubacterium cellulosolvens*) and a Glutamate formimidoyltransferase (homologous to *Prevotella* sp. *HUN102*), among many others (see Supplementary Table S3).

**Fig. 6:**
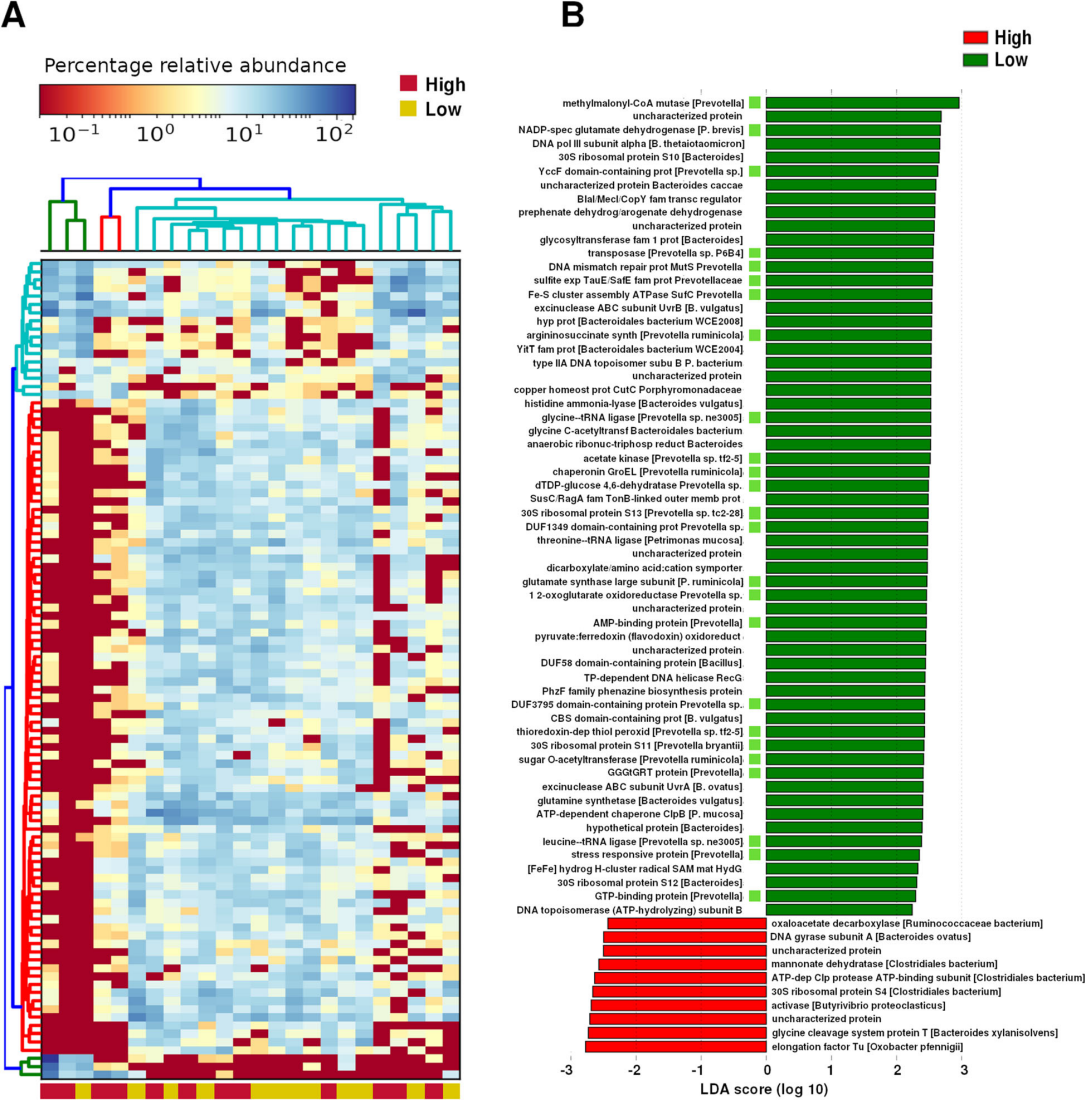
Summary of bacterial protein identification. **a** Hierarchical clustering of the 100 most abundant proteins delineated two clusters of samples (see upper dendrogram). **b** Relative abundance of proteins in animals with high or low methane emissions. Proteins with homology to *Prevotella* proteins are highlighted with green squares

When we conducted hierarchical clustering on the 100 most abundant proteins, the exact same larger cluster of samples identified with Kraken2 taxonomy assignments (Fig. 3a) was recapitulated (Fig. 6a). Based on linear discriminant analysis, 60 enzymes were more abundant in the group with lower emissions of methane, and only 10 were more abundant in the group with higher emissions of methane. Interestingly, 25 enzymes with homology to *Prevotella* proteins are among the hits more abundant in the low-methane-emissions group (Fig. 6b). All those hits, however, were not statistically significant after correction for multiple comparison.

In summary, we conducted a thorough characterization of the rumen microbiome of two small cohorts of buffalos that were found to produce either high or low methane emissions. The main findings suggest increased abundance of *Prevotella* species in the group of low methane emissions. This was very clear in results from several analytical approaches presented here, however statistical significance was not reached in many cases, which probably is derived from intra-group variability. Lack of statistical significance in microbiome means comparisons is often the result of the large number of taxa detected, which makes correction of *p*-values very stringent. The number of animals in each of our experimental groups was relatively small (*n* = 12), which will also result in relatively high p-values. Nonetheless, and apparent higher abundance of *Prevotella* in the group of animals with lower methane emissions is evident.

## Discussion

In this report we present the characterization of the microbiome of two cohorts of Colombian buffalos that produced either high or low methane emissions, in search for microbial determinants of methane production. Our original hypothesis was that a difference in methananogens abundance was the underlying cause of differential methane emissions. We did find subtle differences in four out of five *Methanobacteriaceae* taxa detected in our data. In all four cases, although considerable variability inside groups was evident, animals of the group associated with higher emissions exhibited higher abundance of such methanogens. So, it is possible that subtle differences in methanogens are sufficient to influence methane emissions.

More generally, and in agreement with previous reports, we found that the buffalos microbiome was mainly composed by Bacteroidete*s* and Firmicutes phyla [17, 18, 28]⁠. At the family level, Bacteroidaceae, Lachnospiraceae and F082 predominated, while at the genus level, *Prevotella* and *Butyrivibrio* were dominant. High abundance of *Prevotella* in the rumen microbiome has been reported by several groups in the past [29–33]⁠. Importantly, at all taxonomic levels, the abundance of *Prevotella*, or the upper taxa containing it, were visibly more abundant in the group of animals with lower methane emissions. This effect was more pronounced at the genus level, where 10 out of 12 animal showed high proportion of *Prevotella*.

Taxonomic classification of sequences with Kraken2 [19]⁠ also suggested higher abundance of *Prevotella* in the group with lower methane emissions. This was confirmed by phylogenetic analyses with MAGpy [25]⁠. This approach suggested that *Prevotella ruminicola* and another species in the genus *Prevotella* were more abundant in the group of lower methane emissions. Furthermore, after prediction an annotation of bacterial proteins, 60 enzymes were found to be more abundant in the group of lower methane emissions and from those 25 corresponded to protein sequences with high homology to *Prevotella* proteins. Thus, from these three lines of evidence we conclude that sequences with homology to *Prevotella* were more abundant in the group of animals with lower methane emissions. However, we do acknowledge that after correction of *p*-values for multiple comparisons, most comparisons were statistically non-significant, but the trend observed is very clear and favors the notion that *Prevotella* is more abundant in animals producing less methane. We want to point out that *Prevotella* is likely not the only cause of lower methane emissions and other factors, like animal genotype for example, might also exert an effect on such phenotype, as has been previously suggested [15]⁠.

The above-described observation poses the central question of this discussion. How can we explain reduction in methane emissions based on increased abundance of ruminal *Prevotella*? We favor the hypothesis that increase in the production of propionic acid by *Prevotella* reduces availability of hydrogen for methane production. In different contexts, it has been suggested that hydrogenotrophic bacteria might be used to divert hydrogen away from methane synthesis [10]⁠.

Diet of herbivores consists mostly of complex polysaccharides that are not digestible by host cells and most energetic requirements are satisfied through microbial fermentation [34]⁠. In a simplified manner, polysaccharides breakdown starts with the adhesion of cellulolytic bacteria like *Ruminococcus* and *Fibrobacter* to the substrate. Solubilized polymers, formate, succinate, but also CO_2_ and H_2_, are then intercepted by butyrate-producing bacteria like *Butyrivibrio* and *Roseburia* and succinate- and propionate-producing bacteria like *Bacteroides* and *Prevotella*. Methanogenic archaea compete for the hydrogen pool [34]⁠. Experimentally, it has been demonstrated that adherent fibers and the liquid fraction of the rumen contain similar microbial ensembles, which vary in relative abundance, while the rumen epithelium harbor unique microbial taxa. Namely, adherent fibers are rich in fibrolytic microorganisms like member of the family Ruminococcaceae and the genus *Fibrobacter*, while the aqueous phase have abundant members of Prevotellaceae [35, 36]⁠. Interestingly, in our phylogenetic analyses with MAGpy we found increased abundance of *Fibrobacter*, *Oscillibacter*, *Ruminococcaea bacterium*, *Prevotella*, and *Bacteriodetes bacterium* in the group with lower methane emissions, which perhaps suggests a microbiome with higher fermentative capabilities.

Taxonomy of *Prevotella ruminicola*, one of the best studied species of *Prevotella*, has been revised several times considering microbiological and biochemical evidence. *P. ruminicola* was originally known as *Bacteroides ruminicola* [37]⁠. As far back as 1966, it was demonstrated, using isotopic and enzymatic techniques, that *B. ruminicola* used the acrylate reductive pathway (using acrylyl-CoA as an intermediate) to produce propionate [38]⁠. Not long after, Van Nevel and collaborators elegantly showed that inhibition of methanogenesis by chloral hydrate led to accumulation of gaseous hydrogen and an increase in propionic acid production. It is therefore accepted that metabolic hydrogen produced during ruminal fermentation is partitioned between production of methane, propionic acid and butyric acid [39]⁠.

## Conclusions

We propose that higher abundance of *Prevotella* in the rumen of animals with lower methane emissions negatively influences methane production. More abundant *Prevotella* species would outcompete methanogens for hydrogen utilization, which will be diverted for production of propionic acid.

Of course, our hypothesis needs experimental validation. A simple way to test it would be to measure the content of propionate in animals with high or low abundance of *Prevotella* and methane emissions. Artificial enrichment of *Prevotella* from cultures is also an appealing approach to study metabolic flow. Ultimately, if proven true, microbiota from low methane emission animals could be transferred via ruminal liquid to animals with high production of methane and colonization of the rumen in the latter group would be monitored as well as methane emissions. Transferring the whole microbiome has the obvious advantage that any other microorganism contributing to the hypothesized role of *Prevotella* would also be transferred. Finally, we do not discard a role of genetic factors on methane production. Studies to characterize the genotype in animals with low or high methane production are currently underway.

## Methods

### Description of animals and collection of ruminal samples

The buffalo dairy farm is located at 8**°**10′34″ N and 76**°**03′46″ W in the Tierra Alta locality, in the department of Cordoba, Colombia. The dairy farm contains 11,718 animals, and a database with records that extend back 22 years. For the measurement of CH_4_ emissions, four breathing chambers of metal structure with walls and roof covered with high-density polyethylene and sealed with velcro were used. Dimensions of each chamber were 2.2 m × 1.7 m × 0.9 m length, height and width, respectively, and allowed to control measurement conditions by confining the animals in small spaces and acting as flow accumulators of the gases belched and coming from the excreta. A Gases PRO Sensor Board (Calibrated) from Waspmote (Libelium®) was used, together with a Methane and Fuel Gas Sensor (Calibrated) reference CH-A3, also from Libelium®. The sensor can perform CH_4_ measurements in the environment every 30 s, with a nominal low explosive level (LEL) between zero and 100% with an accuracy of ±0.15% LEL. One buffalo was placed in each of the chambers and the walls were sealed 5 min before the measurement corresponding to each chamber. The measurement was performed individually for each animal for a period of 10 min with captures of CH_4_ in the environment of the breathing chamber by the sensor every 30 s, followed by a measurement of CH_4_ in the environment outside the chamber for a period of 5 min with the same sensor settings. In this way it was possible to obtain the difference between the CH_4_ concentration inside the chamber and the surrounding environment. In total 115 female and six male buffaloes were used to measure methane breath emissions. Each animal was measured three times a day, each measurement lasting 10 min.

Twelve animals, which were found to emit either high (1.36 ± 0.11 g CH_4_/ kg dry matter ingested) or low (− 1.36 ± 0.16 g CH_4_/ kg dry matter ingested) amounts of methane were included in each group.

### Sample collection, DNA extraction, library construction and sequencing

For collection of ruminal samples, animals were sedated with Xylazine (10%). The jaw of animals was immobilized and a 1/2 in. siliconized probe was introduced and ruminal liquid was extracted using a manual Humboldt pump into sterile glass bottles. Samples were then aliquoted into 50 ml Falcon tubes and snap-frozen in liquid nitrogen.

DNA was extracted using the QIAamp PowerFecal DNA Kit (QIAGEN) according to manufacturer’s instructions, which includes bead-beating. DNA was then quantified using Qubit and a dsDNA HS Assay kit (Thermo Fisher Scientific). NexteraXT (Illumina) libraries were constructed from 1 ng of genomic DNA according to manufacturer’s protocols. Indexed libraries were then inspected on a high sensitivity Bioanalyzer 2000 chip (Agilent) and quantified using Qubit as above. For library pooling, molarity of libraries was calculated using the average library size and the DNA concentration and a 4 nM pool was prepared. Libraries were sequenced at 10 pM on a MiSeq instrument (Illumina) using a 300 cycles paired-end protocol that included demultiplexing.

### Bioinformatics analysis

Sequence’s quality was inspected with fastqc and bases with Q scores < 30 were trimmed off with fastq-mcf keeping only sequences with a final length > 100 bases. These are referred to as ‘clean-sequences’ and were used for all downstream procedures.

#### Taxonomic classification

Sequences were classified using Kraken2 [19]. We used the Kraken standard database complemented with all whole-genome and partial sequences of bacteria, archaea, fungi and viruses found in NCBI. Such database was complemented with the Genome Taxonomy Database, GTDB [20] (Fig. 1). Kraken2 results were filtered to allow only hits with at least 10% k-mers aligned.

#### Combined assembly

We conducted combined assembly of all our 24 samples with SPAdes [40]⁠, with default parameters. For deconvolution of assembled contigs, each individual sample was mapped to each contig and the number of aligned reads to each contig was normalized by library size and was considered the relative contribution of each sample to each contig.

#### Metagenome-assembled genome prediction

Contigs generated with SPAdes were also subjected to phylogenetic analysis with MAGpy [25]⁠. Initially, contigs were binned with MetaBAT2 [24]⁠ and such bins were then analyzed with MAGpy. MetaBAT2 generates a file where the relative contribution of each sample to each bin is indicated. We normalized such a table for comparisons of bins after MAGpy annotation.

#### Protein sequences generation

In order to maximize chances of detecting putative bacterial proteins, two complementary approaches were implemented. First, we annotated in silico translated contigs from combined assembly with the software Prokka [41]⁠. Prokka utilizes third-party feature prediction tools to identify genomic features contained in contigs. It translates in silico the features identified and annotates them by comparison with bacterial protein databases. One of the outputs is a FASTA file containing protein sequences predicted from the scaffolds. Second, we used PLASS [42]⁠ to assemble protein sequences de novo. See Fig. 1 for a schematic of our workflow. Since the Prokka translated and the PLASS predicted protein sets may be partially redundant, we concatenated them and conducted clustering with Linclust [43]⁠ to obtain a non-redundant set of representative sequences.

#### Alignments of protein against several databases

We conducted Diamond [44]⁠ alignments against the database UniRef [26]⁠ and against two databases containing ruminal bacterial proteins, UniRef [29]⁠ and Hungate1000 [27]⁠. Because RumiRef includes annotations derived from alignments against multiple databases (CAZy, KEGG, UniRef and Hungate) when possible, we recovered all annotations. To determine the relative contribution of each sample to each protein sequence, each library was aligned with Diamond (method blastx) against the database of non-redundant protein representative sequences and to account for the resolution lost during clustering of protein sequences, a hit was considered true if it had an identity of at least 90% over a stretch of at least 50 amino acids. Moreover, we discarded hits for which less than five reads per sample, on average, were registered and hits that were present in less than three samples in each group.

#### Statistical analysis

In all cases, statistical comparisons were conducted using linear discriminant analyses with the software LEfSe [21]⁠ or by conducting Wilcoxon test in R. A detailed workflow and required scripts describing the implementation of our analysis is publicly available in GitHub (https://github.com/buffGenomic/PipelineColBuff).

## Supplementary Information

**Additional file 1:** **Supplementary Figure S1.** Relative abundance of five *Methanobacteriacea* taxa identified.

**Additional file 2:** **Supplementary Table S1.** Results of taxonomic classification of sequences conducted with Kraken2.

**Additional file 3:** **Supplementary Table S2.** Annotation of MetaBat2 bins and relative abundance in each sample.

**Additional file 4:** **Supplementary Table S3.** Annotation and relative abundance of predicted proteins upon alignments against several protein databases.

## Data Availability

All sequences generated in this study are publicly available from the SRA portal of NCBI under the accession number PRJNA605425.

## Abbreviations

GTDB: Genome Taxonomy Database
NCBI: National Center for Biotechnology Information
MAGpy: A computational pipeline for the downstream analysis of Metagenome-Assembled Genomes (MAGs)
LEL: Low Explosive Level
MTDFREML: Multiple-Trait Derivative -Free REstricted Maximum Likelihood
Qscore: Quality score
SPAdes: A genome assembly algorithm
MetaBAT2: Software to bin contigs
Prokka: Software for prokaryotic genome annotation
PLASS: Protein-Level ASSembler
UniRef: Comprehensive and non-redundant UniProt reference clusters
RumiRef: Reference database of protein clusters identified in the rumen microbiome
CAZy: Database of Carbohydrate-Active enZYmes.
KEGG: Kyoto Encyclopedia of Genes and Genomes
Blastx: An algorithm to search protein databases using a translated nucleotide query
LEfSe: Linear discriminant analysis Effect Size
GitHub: A portal that provides hosting for software development and version control using Git
NGS: Next Generation Sequencing
HUMANn2: HMP Unified Metabolic Analysis Network
SRA: Sequence Read Archive
Hungate1000: Reference set of rumen microbial genome sequences

## References

[1] Tian H, Lu C, Ciais P, Michalak AM, Canadell JG, Saikawa E (2016). The terrestrial biosphere as a net source of greenhouse gases to the atmosphere. Nature..

[2] Huang J, Yu H, Dai A, Wei Y, Kang L (2017). Drylands face potential threat under 2 °c global warming target. Nat Clim Chang.

[3] Kirschke S, Bousquet P, Ciais P, Saunois M, Canadell JG, Dlugokencky EJ (2013). Three decades of global methane sources and sinks. Nat Geosci.

[4] Nisbet EG, Dlugokencky EJ, Bousquet P (2014). Methane on the rise - again. Science..

[5] Huws SA, Creevey CJ, Oyama LB, Mizrahi I, Denman SE, Popova M (2018). Addressing global ruminant agricultural challenges through understanding the rumen microbiome: Past, present, and future. Front Microbiol.

[6] Wollenberg E, Richards M, Smith P, Havlík P, Obersteiner M, Tubiello FN (2016). Reducing emissions from agriculture to meet the 2 °C target. Glob Chang Biol.

[7] Hook SE, Wright ADG, McBride BW. Methanogens: methane producers of the rumen and mitigation strategies. Archaea. 2010;2010. 10.1155/2010/945785 PMC3021854 21253540

[8] Matthews C, Crispie F, Lewis E, Reid M, O’Toole PW, Cotter PD (2019). The rumen microbiome: a crucial consideration when optimising milk and meat production and nitrogen utilisation efficiency. Gut Microbes.

[9] Thauer RK, Kaster AK, Seedorf H, Buckel W, Hedderich R (2008). Methanogenic archaea: ecologically relevant differences in energy conservation. Nat Rev Microbiol.

[10] Lan W, Yang C (2019). Ruminal methane production: associated microorganisms and the potential of applying hydrogen-utilizing bacteria for mitigation. Sci Total Environ.

[11] Söllinger A, Tveit AT, Poulsen M, Noel SJ, Bengtsson M, Bernhardt J, et al. Holistic Assessment of Rumen Microbiome Dynamics through Quantitative Metatranscriptomics Reveals Multifunctional Redundancy during Key Steps of Anaerobic Feed Degradation. mSystems. 2018;3. 10.1128/mSystems.00038-18 PMC6081794 30116788

[12] Tapio I, Snelling TJ, Strozzi F, Wallace RJ. The ruminal microbiome associated with methane emissions from ruminant livestock. J Animal Sci Biotechnology. 2017;8. 10.1186/s40104-017-0141-0 PMC5244708 28123698

[13] John Wallace R, Sasson G, Garnsworthy PC, Tapio I, Gregson E, Bani P, et al. A heritable subset of the core rumen microbiome dictates dairy cow productivity and emissions. Sci Adv. 2019;5. 10.1126/sciadv.aav8391 PMC6609165 31281883

[14] Li F, Li C, Chen Y, Liu J, Zhang C, Irving B, et al. Host genetics influence the rumen microbiota and heritable rumen microbial features associate with feed efficiency in cattle. Microbiome. 2019;7. 10.1186/s40168-019-0699-1 PMC6567441 31196178

[15] Difford GF, Plichta DR, Løvendahl P, Lassen J, Noel SJ, Højberg O (2018). Host genetics and the rumen microbiome jointly associate with methane emissions in dairy cows. PLoS Genet.

[16] Singh KM, Reddy B, Patel AK, Panchasara H, Parmar N, Patel AB (2014). Metagenomic analysis of buffalo rumen microbiome: effect of roughage diet on dormancy and sporulation genes. Meta Gene.

[17] Kala A, Kamra DN, Kumar A, Agarwal N, Chaudhary LC, Joshi CG (2017). Impact of levels of total digestible nutrients on microbiome, enzyme profile and degradation of feeds in buffalo rumen. PLoS One.

[18] Zou C, Gu Q, Zhou X, Zhongsheng X, Muhammad WI, Tang Q (2019). Ruminal microbiota composition associated with ruminal fermentation parameters and milk yield in lactating buffalo in Guangxi, China—A preliminary study. J Anim Physiol Anim Nutr (Berl).

[19] Wood DE, Lu J, Langmead B (2019). Improved metagenomic analysis with kraken 2. Genome Biol.

[20] Chaumeil P-A, Mussig AJ, Hugenholtz P, Parks DH. GTDB-Tk: a toolkit to classify genomes with the genome taxonomy database. Bioinformatics. 2019. 10.1093/bioinformatics/btz848. 10.1093/bioinformatics/btz848 PMC7703759 31730192

[21] Segata N, Izard J, Waldron L, Gevers D, Miropolsky L, Garrett WS, et al. Metagenomic biomarker discovery and explanation. Genome Biol. 2011;12. 10.1186/gb-2011-12-6-r60 PMC3218848 21702898

[22] Franzosa EA, McIver LJ, Rahnavard G, Thompson LR, Schirmer M, Weingart G (2018). Species-level functional profiling of metagenomes and metatranscriptomes. Nat Methods.

[23] Jovel J, Patterson J, Wang W, Hotte N, O’Keefe S, Mitchel T, et al. Characterization of the gut microbiome using 16S or shotgun metagenomics. Front Microbiol. 2016;7. 10.3389/fmicb.2016.00459 PMC4837688 27148170

[24] Kang DD, Li F, Kirton E, Thomas A, Egan R, An H, et al. MetaBAT 2: An adaptive binning algorithm for robust and efficient genome reconstruction from metagenome assemblies. PeerJ. 2019;2019. 10.7717/peerj.7359 PMC6662567 31388474

[25] Stewart RD, Auffret MD, Snelling TJ, Roehe R, Watson M (2019). MAGpy: a reproducible pipeline for the downstream analysis of metagenome-assembled genomes (MAGs). Bioinformatics..

[26] Suzek BE, Wang Y, Huang H, McGarvey PB, Wu CH (2015). UniRef clusters: a comprehensive and scalable alternative for improving sequence similarity searches. Bioinformatics..

[27] Seshadri R, Leahy SC, Attwood GT, Teh KH, Lambie SC, Cookson AL (2018). Cultivation and sequencing of rumen microbiome members from the Hungate1000 collection. Nat Biotechnol.

[28] Singh KM, Ahir VB, Tripathi AK, Ramani UV, Sajnani M, Koringa PG (2012). Metagenomic analysis of Surti buffalo (Bubalus bubalis) rumen: a preliminary study. Mol Biol Rep.

[29] Stewart RD, Auffret MD, Warr A, Walker AW, Roehe R, Watson M (2019). Compendium of 4,941 rumen metagenome-assembled genomes for rumen microbiome biology and enzyme discovery. Nat Biotechnol.

[30] Stewart RD, Auffret MD, Warr A, Wiser AH, Press MO, Langford KW, et al. Assembly of 913 microbial genomes from metagenomic sequencing of the cow rumen. Nat Commun. 2018;9. 10.1038/s41467-018-03317-6 PMC5830445 29491419

[31] Matsui H;, Ogata K, Tajima K, Nakamura M, Nagamine T, Aminov R, et al. Phenotypic characterization of polysaccharidases produced by four *Prevotella* type strains. Curr Microbiol 2020;41:45. 10.1007/s002840010089 10919398

[32] Stevenson DM, Weimer PJ. Dominance of *Prevotella* and low abundance of classical ruminal bacterial species in the bovine rumen revealed by relative quantification real-time PCR. Appl Microbiol Biotechnol 2007;75:165–174. 10.1007/s00253-006-0802-y 17235560

[33] Fondevila M, Dehority BA. Interactions between *Fibrobacter succinogenes*, *Prevotella* ruminicola, and *Ruminococcus flavefaciens* in the digestion of cellulose from forages. J Anim Sci 1996;74:678–684. 10.2527/1996.743678x 8707727

[34] Flint HJ, Bayer EA, Rincon MT, Lamed R, White BA (2008). Polysaccharide utilization by gut bacteria: potential for new insights from genomic analysis. Nat Rev Microbiol.

[35] Vaidya JD, van den Bogert B, Edwards JE, Boekhorst J, van Gastelen S, Saccenti E, et al. The effect of DNA extraction methods on observed microbial communities from fibrous and liquid rumen fractions of dairy cows. Front Microbiol. 2018;9. 10.3389/fmicb.2018.00092 PMC5797766 29445366

[36] Deusch S, Camarinha-Silva A, Conrad J, Beifuss U, Rodehutscord M, Seifert J (2017). A structural and functional elucidation of the rumen microbiome influenced by various diets and microenvironments. Front Microbiol.

[37] Avguštin G, Wallace RJ, Flint HJ. Phenotypic diversity among ruminal isolates of *Prevotella* ruminicola: proposal of *Prevotella* brevis sp. nov., *Prevotella bryantii* sp. nov., and *Prevotella albensis* sp. nov. and redefinition of *Prevotella ruminicola*. Int J Syst Bacteriol 1997;47:284–288. 10.1099/00207713-47-2-284 9103611

[38] Wallnöfer P, Baldwin RL. Pathway of propionate formation in *Bacteroides ruminicola*. J Bacteriol 1967;93:504–505. http://www.ncbi.nlm.nih.gov/pubmed/6020420. Accessed 12 Feb 2020. 10.1128/jb.93.1.504-505.1967 PMC315028 6020420

[39] Van Nevel CJ, Henderickx HK, Demeyer DI, Martin J (1969). Effect of chloral hydrate on methane and propionic acid in the rumen. Appl Microbiol.

[40] Bankevich A, Nurk S, Antipov D, Gurevich AA, Dvorkin M, Kulikov AS (2012). SPAdes: a new genome assembly algorithm and its applications to single-cell sequencing. J Comput Biol.

[41] Seemann T (2014). Prokka: rapid prokaryotic genome annotation. Bioinformatics..

[42] Steinegger M, Mirdita M, Söding J (2019). Protein-level assembly increases protein sequence recovery from metagenomic samples manyfold. Nat Methods.

[43] Steinegger M, Söding J. Clustering huge protein sequence sets in linear time. Nat Commun. 2018;9. 10.1038/s41467-018-04964-5 PMC6026198 29959318

[44] Buchfink B, Xie C, Huson DH (2014). Fast and sensitive protein alignment using DIAMOND. Nat Methods.

