# Supplementary figures and images for: Lower methane emissions were associated with higher abundance of ruminal Prevotella in a cohort of Colombian buffalos
Source: BMC Microbiol. 2020 Nov 27;20:364. doi: 10.1186/s12866-020-02037-6 (PMC7694292; doi:10.1186/s12866-020-02037-6)

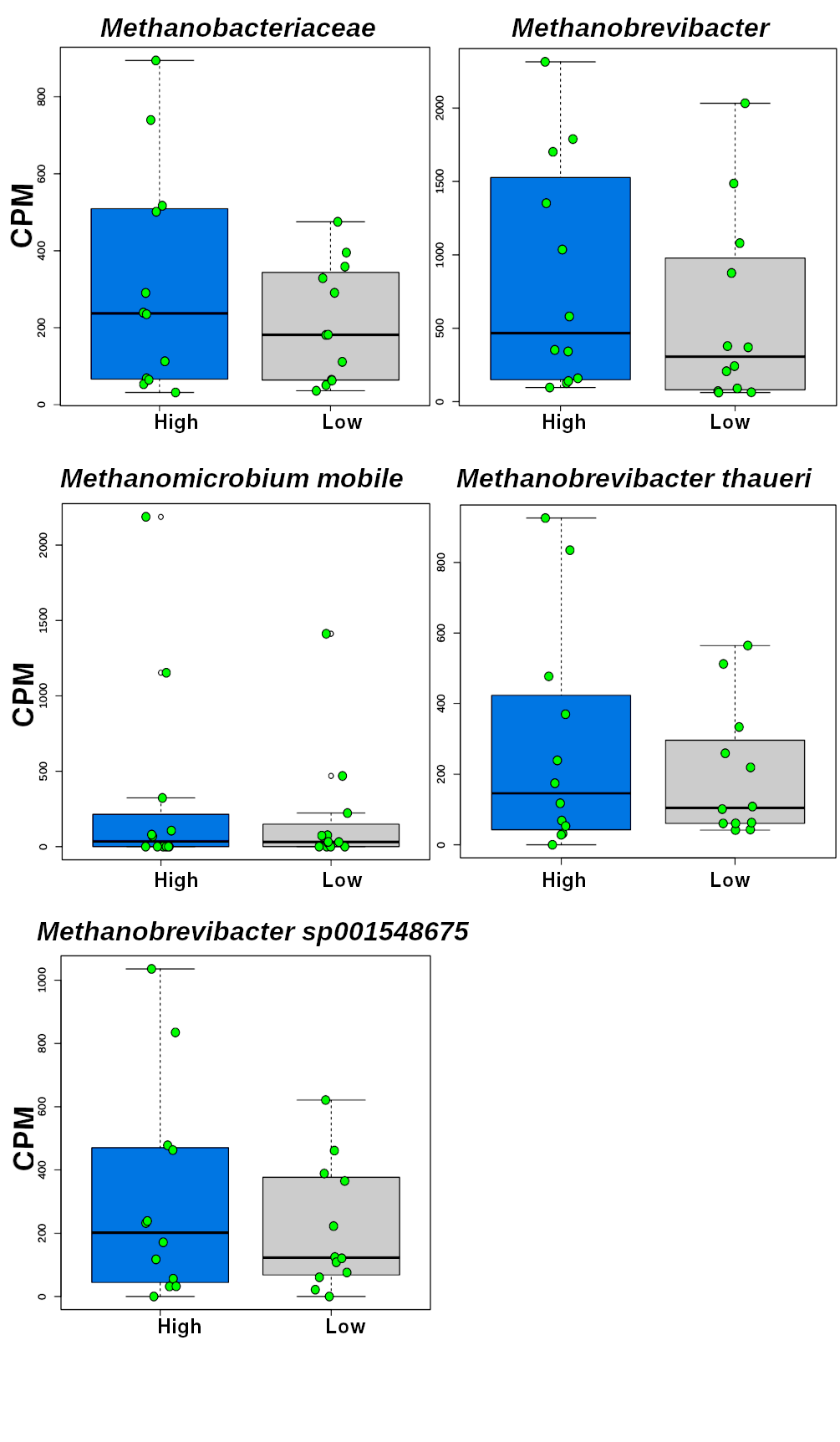

Supplement: Supplementary file 1 — Additional file 1: Supplementary Figure S1. Relative abundance of five Methanobacteriacea taxa identified. [file 12866_2020_2037_MOESM1_ESM.png]
